# Grand Challenge in Human/Animal Virology: Unseen, Smallest Replicative Entities Shape the Whole Globe

**DOI:** 10.3389/fmicb.2020.00431

**Published:** 2020-03-18

**Authors:** Akio Adachi

**Affiliations:** ^1^Department of Microbiology, Kansai Medical University, Osaka, Japan; ^2^Tokushima University, Tokushima, Japan

**Keywords:** human viruses, animal viruses, pathogenic viruses, virology, biology, multidisciplinary science

By far the most abundant species in nature is the virus that cannot replicate by itself. Viruses, parasitic entities, are found in virtually all unicellular and multicellular creatures. Viruses are everywhere. While smallest in size among all species, they can ingeniously replicate, persist, and survive in their individual hosts and/or host populations, and are transmittable among hosts. Viruses are sometimes inflicting or fatal for host species and are sometimes inter-species replicons. They keep interacting with their hosts in numerous different manners. Unseen viruses thus can reshuffle the whole world through accumulations of their subtle biological effects. In other words, viruses can shape the entire environment around us and are able to directly and/or indirectly influence us by their biologic activities. Virology is a multidisciplinary research field and, as an academic discipline of the biology, it extensively analyzes all aspects of viruses derived from every living species by scientific systems/methodologies currently available to us as exemplified and fully described in a series of Frontiers special issues designated “Research Topic” (RT) in the Virology section of Frontiers in Microbiology (Nomaguchi and Adachi, 2010, 2017; Miyazaki et al., 2012; Nomaguchi et al., 2012; Berkhout and Coombs, 2013; Sato et al., 2013; Adachi and Miura, 2014; Dutilh et al., 2017; Sanfaçon, 2017; Yamamoto et al., 2017). I wish to emphasize here again that virology is a branch of biological sciences studying the fundamental attributes of a wide variety of unique characteristic viruses. Virology concerns biological issues in a broad sense.

In the past decade, as many as of 1,500 articles approximately have been published and almost 80 RTs for specific subjects have been issued or are on the way in the Virology section. During this period, we have seen constant and vast increases both in virology-specific, in a narrow sense, articles, and in those giving more conceptual and general knowledge of the biology. Notably, the observed advances frequently have been accompanied by remarkable development of novel innovative technologies. In the next decade, concomitant with successful proceeding of the powerful and sharp analyzing systems and/or methods such as computational biology, structural biology, bioinformatics, multi-omics, next-generation sequencing, genome editing, single-cell methodology, and organoid technology (Bai et al., 2015; Angermueller et al., 2016; Chen et al., 2017; Dutta et al., 2017; Hasin et al., 2017; Adli, 2018; Artegiani and Clevers, 2018; Cheng, 2018; Ibrahim et al., 2018; Rossi et al., 2018; Sbalzarini and Greber, 2018; van Dijk et al., 2018; Pickar-Oliver and Gersbach, 2019), virology will certainly continue to contribute much to the progress in all areas of the basic biology from molecular and structural biology to environmental science. In addition, virology will surely represent one of major driving forces to solve a wide variety of practical issues, which are directly or indirectly related to the virus issue itself, i.e., nanotechnology, viral vectors, antiviral drugs, vaccines, gene therapy, interferon therapy, immune therapy, and so on (De Clercq, 2007; Guimarães et al., 2015; Szunerits et al., 2015; De Clercq and Li, 2016; Athanasopoulos et al., 2017; Grimm and Büning, 2017; Singh et al., 2017; Snell et al., 2017; Chambers et al., 2018; Colino et al., 2018; Kaufmann et al., 2018; Ono et al., 2018; Sulczewski et al., 2018; Vetter et al., 2018; Dionne, 2019; Graham et al., 2019). It is well-expected from our plentiful experience in the past decade that the Virology section, as an outstanding communication platform, plays a critical and central role in various activities of basic and applied sciences.

Human and animal virology investigates viruses that infect human and all species of animals. Upon replication, some viruses are damaging for their hosts to various degrees whereas others rather peacefully co-exist with the hosts. Among these viruses, those that cause serious infectious diseases in human and/or animals, are medically, socially, and economically of particular importance, as a matter of course for scientific significance. In fact, our Virology section has published numerous articles related to this kind of human and animal viruses and has edited relevant RTs in the past decade. On one hand, more general subjects covering various pathogenic viruses and related research areas have been targeted as well. Of socially important pathogenic viruses, some are solely tropic for humans, HIV-1 as an example (Hatziioannou et al., 2006, 2009; Kamada et al., 2006; Nomaguchi et al., 2008, 2013b), and some like human norovirus are known not to replicate in cultured cells (Duizer et al., 2004; Herbst-Kralovetz et al., 2013; Ettayebi et al., 2016; Murakami et al., 2020). Indeed, these viral properties hamper and limit our experimental strategies, and are representing major study projects to overcome in the virology. Alternative practical experimental systems, irrespective of being commonly useful and applicable to numbers of viruses or specifically to certain viruses, to circumvent the observed difficulties are definitely required. Expert researchers on the viruses have been making every effort to achieve the primary purposes (Kamada et al., 2006; Nomaguchi et al., 2011, 2013b; Soll et al., 2013; Hatziioannou et al., 2014; Ettayebi et al., 2016; Doi et al., 2018; Schmidt et al., 2019; Murakami et al., 2020). The trend described above is understandable and well-recognized by published articles and RTs in the human and animal virus field in the Virology section. Most cited articles and most viewed RTs in the human/animal virus field of the section (top 5, as of February 3, 2020) are as follows, respectively: *articles*, “Epidemiological aspects and world distribution of HTLV-1 infection” (Gessain and Cassar, 2012), “Pathology of asthma” (Kudo et al., 2013), “Challenges and opportunities in estimating viral genetic diversity from next-generation sequencing data” (Beerenwinkel et al., 2012), “ER stress, autophagy, and RNA viruses” (Jheng et al., 2014), and “Zika virus: the latest newcomer” (Saiz et al., 2016); *RTs*, “Highly mutable animal RNA viruses: adaptation and evolution” (Nomaguchi and Adachi, 2017), “Virus discovery by metagenomics: the (im)possibilities” (Dutilh et al., 2017), “Zika virus research” (Bueno-Marí et al., 2018), “Pathophysiology and epidemiology of virus-induced asthma” (Kimura and Ryo, 2014), and “Forefront studies on HTLV-1 oncogenesis” (Mahieux and Watanabe, 2013). The results described above may indicate the research field in which our peers are mostly interested. Another point worth mentioning here is the emerging pathogenic viruses such as hemorrhagic Ebola virus and pneumonia-causing new coronavirus designated SARS-CoV-2. These viruses are becoming more and more important under today's world environment, as is the case for the influenza virus, a continuous global health threat, that potentially can induce an awful pandemic. It is more and more probable that humans may encounter emerging zoonotic viral pathogens and also re-emerging viruses through extensive inroads into untouched areas. We of course do not know much about the biology, including the ecology, origin, mutation, adaptation, etc., of these new viruses as yet. Once transmitted to humans, they readily replicate in and spread among human populations without effective immunity against them. Care should be taken not to be infected with the viruses at individual and population levels. Infected individuals must be treated properly. In this regard, we virologists always have to prepare the ground for sudden onsets of new emerging diseases. Routine collaborative research activities among basic researchers, clinical doctors, and relevant staffs of various expertise underpin the basis for swift responses against them. Valid anti-viral strategies can be generated by such well-organized medical teams.

Viruses can cause acute or chronic infections in human and animal hosts in many mechanistically different ways. While causative viruses for the former do not persist long in individual hosts in general, the latter viruses generate persistent infection and may give rise to latent infection. It is scientifically and practically important and of great interest to determine the underlying mechanisms. Elucidating biological and molecular bases for the conflict between viruses and their hosts is therefore one of major missions of current virology. Especially, for some pathogenic viruses that have incredibly lengthy interacting period with hosts, or that are prone to readily alter in nature, it is critically important to dynamically investigate their mutations, adaptation, and evolution. HIV-1, a representative virus for such category, readily mutates and adapts itself in the course of infection as experimentally revealed by a series of analytical studies (Nomaguchi et al., 2008, 2013a,c, 2014, 2016, 2017, 2018; Saito et al., 2011; Yokoyama et al., 2016; Doi et al., 2019). Here, following seven RTs in our Virology section are cited as examples of various cases for viral interactions with their hosts: “Codon usage and dinucleotide composition of virus genomes: from the virus-host interaction to the development of vaccines,” “Host and pathogen mechanisms underpinning viral ecology and emerging infections,” “Revealing HIV hiding places: identification and characterization of cellular and tissue reservoirs,” “The interplay between innate immunity and herpesviruses,” “HIV-1 genetic diversity,” “Highly mutable animal RNA viruses: adaptation and evolution” (Nomaguchi and Adachi, 2017), and “The past and the future of human immunity under viral evolutionary pressure” (Hurst and Magiorkinis, 2019). As can be realized by the brief descriptions for the RTs and articles published in the RTs, there are indeed numerous distinct virus-host interactions to be noted. Lastly, regardless of their medical or economic effects on human society, all RNA and DNA viruses are equally valid targets for extensive scientific studies in today's virology. We aim to publish important articles resulting from those studies that significantly advance our understanding of the viruses and related issues in our Virology section.

The most noticeable topic in the field of human and animal virology in the next decade would depend upon what happens in the virological world around us. It could be a deadly virus that causes global infection, or, could be some discovery or invention that changes our concept. Attention should be paid to endogenized viral elements that affect the hosts in a biologically significant way. The relevant research field is rapidly growing as described recently in our journal (Staege and Emmer, 2018; Turnbull and Douville, 2018; Flynn and Moreau, 2019; Moelling and Broecker, 2019). As for the correct answer to the question above, it is of course unpredictable at the present time but the virological information and biological finding of the first magnitude that move the academic and public communities will surely determine the result. Based on enormous accumulations of scientific and statistical data of numerous kinds in various fields, together with orthodox and innovative analytical systems, it is quite expected that future virology can further deepen studies on virus populations and predictive science as well as those on a specific individual virus. From this point of view, I would like to enthusiastically encourage researchers outside the virology field, in addition to those who inside, to submit their representative works to our Virology section, if there is a little relevance. Virology is multidisciplinary in its marked and essential characteristic as stated above. Nevertheless, the bottommost role for current virology in the era of “Big Data” is to functionally/biologically characterize individual viruses for the future by all available tools. By focusing on viruses, we can study and analyze living replicating creatures in all directions. We the Virology section of Frontiers in Microbiology most welcome the collaborative and integrative studies that impact and stimulate the whole world. Join us and share the fruits of virology.

## Author Contributions

AA is a sole contributor to this manuscript and approved its submission.

### Conflict of Interest

The author declares that the research was conducted in the absence of any commercial or financial relationships that could be construed as a potential conflict of interest.
